# Herbal Cuscutae Semen Contributes to Oxidative Stress Tolerance and Extends Lifespan via Sirtuin1 in *Caenorhabditis elegans*

**DOI:** 10.3390/antiox14070786

**Published:** 2025-06-26

**Authors:** Chunyan Chen, Yudie Liu, Jing Hu, Yihan Gu, Weiwei Li, Hui Yue, Sijing An, Na Sun, Peng Zhang, Nan Li, Lin Miao

**Affiliations:** 1 Key Laboratory of Pharmacology of Traditional Chinese Medical Formulae, Ministry of Education, Tianjin University of Traditional Chinese Medicine, Tianjin 301617, China; 18848820783@163.com (C.C.); huujing0438@163.com (J.H.); hanhan030225@163.com (Y.G.); v599vl@163.com (W.L.); yuehui0606@163.com (H.Y.); ansj10063@tjutcm.edu.cn (S.A.); sunna1020@tjutcm.edu.cn (N.S.); zhangpeng@tjutcm.edu.cn (P.Z.); 2 State Key Laboratory of Component-Based Chinese Medicine, Tianjin University of Traditional Chinese Medicine, Tianjin 301617, China; lyd1530781677@163.com; 3 State Key Laboratory of Chinese Medicine Modernization, Tianjin University of Traditional Chinese Medicine, Tianjin 301617, China

**Keywords:** Cuscutae Semen, anti-aging, silent information regulator sirtuin 1, mitochondrial oxidative stress, *Caenorhabditis elegans*

## Abstract

Cuscutae Semen (CS), a traditional herb recognized as a nutraceutical food in China, has been widely utilized in managing aging-related diseases throughout history. However, whether this mechanism is associated with mitochondrial stress tolerance remains unclear. In the present study, *Caenorhabditis elegans* (*C. elegans*) was used to investigate the effects of CS on their longevity. The data demonstrated that CS prolonged the average lifespan of the nematodes by 15.26%, reducing lipofuscin accumulation by 61.46%, as well as improving spontaneous motility. CS treatment significantly enhanced the resistance of *C. elegans* to hydrogen peroxide-induced oxidative stress and 37 °C induced heat stress, reducing reactive oxygen species (ROS) production by 71.45%. Additionally, membrane potential (MMP) and adenosine triphosphate (ATP) were increased by 354.72% and 69.64%, respectively. However, mitochondrion-specific ROS and calcium flux were significantly reduced to 45.86% and 63.25%, respectively, in *C. elegans* treated with CS. Consistently, the polymerase chain reaction data revealed that CS significantly up-regulated the expressions of the antioxidant-related genes *skn-1*, *ctl-1*, *sod-3*, and *gst-4*; the heat shock gene *hsp-16.2*; and the autophagy-related genes *lgg-1* and *bec-1*. Considering the crucial role of the silent information regulator sirtuin 1 (SIR-2.1/SIRT1) in aging-related mitochondrial oxidative stress, we examined its expression and transcriptional activity. As expected, treatment with CS induced SIRT1 expression, and isorhamnetin identified from CS extract significantly enhanced SIRT1 transcriptional activity in HEK293T cells. Collectively, our results provided evidence that CS prolonged the lifespan of *C. elegans* by ameliorating oxidative stress damage and mitochondrial dysfunction via SIRT1.

## 1. Introduction

Aging is a major risk factor for the onset and progression of age-related diseases. Additionally, aging is not only an irreversible biological process characterized by a degenerative decline in physiological functions and diminished adaptability and resistance; but also presents a challenge in the search for anti-aging candidates to delay its progression [1,2]. Oxidative stress has been well accepted as one of the critical causes of aging, contributing to mitochondrial dysfunction, including the loss of mitochondrial membrane potential (MMP) and adenosine triphosphate (ATP) activity [3,4]. Therefore, targeting antioxidant defenses and mitochondrial homeostasis is crucial for addressing aging and age-associated diseases.

*Caenorhabditis elegans* (*C. elegans*) is an ideal model organism widely used in aging studies due to its short lifespan and high fertility [5,6,7]. *C. elegans* is estimated to possess nearly 20,000 protein-coding genes, approximately 60–80% of which are homologous to human genes involved in aging, apoptosis, metabolism, signal transduction, and cell cycling [8]. Various oxidative stress signaling pathways, including insulin/insulin-like growth factor signaling-1 (IIS), heat shock protein (HSP), and mitochondrial pathways, have also been identified in *C. elegans* [7,9,10]. The silent information regulator sirtuin (sirtuin) is a highly conserved group of nicotinamide adenine dinucleotide-dependent deacetylases, with SIR-2.1/SIRT1 serving as a key regulator of the aging process in nematodes [11,12]. The overexpression of *sir-2.1* in *C. elegans* extends lifespan, whereas deletion or knockdown shortens lifespan [12,13,14]. The activation of SIRT1 deacetylates its downstream factors, such as nuclear factor erythroid 2-related factor 2 (SKN-1/NRF2), forkhead box (DAF-16/FOXO), and heat shock (HSF-1/HSF1), enhancing their stability and activity [15,16]. SKN-1 is a homolog of the mammalian NRF2 protein that is deacetylated by SIRT1 to protect against oxidative damage and inhibit apoptosis and inflammatory responses [15,17]. In *C. elegans*, overexpressing of the sirtuin gene *sir-2.1* potentially activates DAF-16/FOXO, which directly protects against oxidative stress through deacetylation and extends lifespan [10,14]. During thermal stress, HSF1 activity is modulated by SIR-2.1/SIRT1-mediated deacetylation [18]. Activated HSF1 binds to HSEs, up-regulating molecular chaperones (e.g., HSP-16.2), and subsequently enhancing thermotolerance [19].

The traditional Chinese medicine Cuscutae Semen (CS), also known as Chinese Dodder or Tu-Si-Zi, is the mature dried seed of *Cuscuta australis* R. Br and *Cuscuta chinensis* Lam [20,21]. As a traditional Chinese medicine with dual roles as both medicine and food, CS exhibits extensive pharmacological activities [22,23,24]. Pharmacological researchers have reported the antioxidant activity of CS against anti-aging [25,26,27,28]. Additionally, in vitro studies have confirmed that CS exerts protective effects against mitochondrial dysfunction via SIRT1 [29,30,31]. However, whether the role of CS in aging is related to SIRT1 expression and mitochondrial protection remains unexplored. In our study, the lifespan and stress resistance of *C. elegans* were investigated, and the mechanisms and active compound(s) involved were determined.

## 2. Materials and Methods

### 2.1. Materials

Peptone (cat. no. P8450), agar powder (cat. no. A8190), sodium chloride (cat. no. S8211), potassium dihydrogen phosphate (cat. no. P7390), dipotassium hydrogen phosphate (cat. no. D8490), cholesterol (cat. no. C8280), magnesium sulfate (cat. no. M9400), calcium chloride (cat. no. C7250), and LB broth (cat. no. L1010, powder) were purchased from Solarbio Science & Technology Co., Ltd. (Beijing, China). Sodium hydroxide (S817979) and sodium hypochlorite solutions (767470, 10% *w*/*v* in water) were purchased from Macklin Inc. (Shanghai, China). Caffeic acid (cat. no. B20660, high-performance liquid chromatography (HPLC ≥ 98%), hyperoside (cat. no. B20631, HPLC ≥ 98%), quercetin (cat. no. B20527, HPLC ≥ 98%), chlorogenic acid (cat. no. wkq-0000358, HPLC ≥ 98%), isorhamnetin (cat. no. wkq-0000665, HPLC ≥ 98%), cryptochlorogenic acid (cat. no. wkq-0000930, HPLC ≥ 98%), and citric acid (Cat. No. wkq-0000891, HPLC ≥ 98%) were supplied from Shanghai yuanye Bio-Technology Co., Ltd. (Shanghai, China) and Sichuan Victory Biotechnology Co., Ltd. (Chengdu, China).

### 2.2. CS Extract Preparation

CS was purchased from Bozhou Lier Chinese Herbal Medicine Slices Company (Bozhou, China). The dried CS material was soaked thrice in 70% (*v*/*v*) ethanol for 24 h each. The filtered extract was concentrated under reduced pressure and lyophilized to obtain CS extract powder.

### 2.3. C. Elegans Strains and Maintenance

Wild-type N2 and mutant VC199 (*sir-2.1(ok434) IV*) strains were provided by the Caenorhabditis Genetics Center (University of Minnesota, USA), which was funded by the National Institutes of Health Office of Research Infrastructure Programs (P40 OD011440). *C. elegans* strains were maintained at 20 °C on nematode growth medium (NGM) plates seeded with a live fresh lawn of *Escherichia coli* (*E. coli*) OP50 as the food source. For subsequent assays, an age-synchronized nematode population was obtained using a standard hypochlorite bleaching method. Nematodes were synchronized to the L4 stage and incubated with concentrated 5-fold *E. coli* OP50 in Petri dishes. Furthermore, CS was prepared in *E. coli* OP50 at final concentrations of 1.25, 2.5, and 5 mg/mL starting from a 10 mg/mL stock solution.

### 2.4. Food Clearance Assay

Approximately thirty L4 synchronized nematodes were transferred to culture medium containing varying concentrations of CS extracts (0, 1.25, 2.5, 5 mg/mL) with a 5-fold concentration of *E. coli* OP50, and incubated at 20 °C for 5 d in a 12-well plate. Absorbance at 595 nm was measured daily.

### 2.5. Lifespan Assay

The lifespan assay was performed as previously described [32]. In brief, approximately one hundred L4-stage nematodes were placed on NGM plates seeded with fresh *E. coli* OP50 containing CS (0, 2.5, 5 mg/mL) for 5 d. This point was defined as the starting time point (day 0) of the lifespan assay. During the initial 5-day drug treatment, nematodes were transferred daily to fresh agar plates, then moved to NGM plates without CS every other day until all the nematodes died. All nematodes were observed under a stereoscopic microscope (ZEISS, Jena, Germany) to record survival and death.

### 2.6. Body Bending Assay

After synchronization, nematodes were treated with CS (0, 2.5, 5 mg/mL) following the lifespan assay protocol and incubated for 5 d. Approximately thirty nematodes were randomly selected, and the number of body bends within 30 s was counted.

### 2.7. Pharyngeal Pumping Assay

After 5 or 10 d of adulthood, pharyngeal pumping was quantified by recording the movement of the pharyngeal bulb at 60 s intervals. For each measurement, thirty nematodes were randomly selected from each population.

### 2.8. Lipofuscin Assay

On day 10 of adulthood, L4-stage nematodes were washed with M9 buffer and transferred to new NGM plates without *E. coli* OP50. Approximately thirty nematodes were mounted on slides containing 0.01% levamisole hydrochloride (L8230, Solarbio, Beijing, China) to visualize intestinal fluorescence under a fluorescence microscope (Leica, Wetzlar, Germany). Lipofuscin levels were quantified using ImageJ v1.8.0 software to determine the fluorescence intensity.

### 2.9. Fecundity Assay

L4-stage nematodes were randomly selected and cultured on NGM plates containing CS (0, 2.5, 5 mg/mL). The parental nematodes were transferred to fresh plates daily until reproduction ceased. Additionally, the number of hatched eggs was counted daily.

### 2.10. Oxidative or Heat Stress Resistance Assay

The L4-stage nematodes were randomly transferred to plates containing different concentrations of CS and incubated for 5 d. Subsequently, the nematodes of each group were placed in *E. coli* OP50 containing 2 mM hydrogen peroxide (H_2_O_2_) (323381, Sigma-Aldrich, St. Louis, MO, USA) or placed in *E. coli* OP50 at 37 °C. The number of dead nematodes per hour was recorded until all nematodes died.

### 2.11. Quantification of Malondialdehyde (MDA)

The nematodes incubated with CS (0, 2.5, 5 mg/mL) for 5 d were collected, and the production of MDA was determined by assessing the optical density according to the manufacturer’s protocol (G0109W, Grace Biotechnology, Qingdao, China) using a Spark multimode microplate reader (Tecan, Grodig, Austria). The protein concentration was determined using the bicinchoninic acid assay. The MDA levels were normalized to the protein concentration in each group.

### 2.12. Quantification of Antioxidant Enzyme Activity

L4-stage nematodes incubated with CS (0, 2.5, 5 mg/mL) for 5 d were collected and sonicated. The lysate was then centrifuged at 15,000 rpm, 4 °C for 10 min, and the supernatant was collected. The levels of superoxide dismutase (SOD, G0101W, Grace Biotechnology, China), catalase (CAT, G0105W, Grace Biotechnology, China), glutathione (GSH, G0206W, Grace Biotechnology, China), or total antioxidant capacity (T-AOC, G0115W, Grace Biotechnology, China) were determined according to the manufacturer’s instructions.

### 2.13. ATP Determination

Approximately fifty L4-stage nematodes were harvested and sonicated after incubation with 2.5 or 5 mg/mL CS for 5 d. The sonicated lysate was centrifuged at 12,000 rpm, 4 °C for 10 min. The supernatant was collected, and the ATP content was determined using an ATP assay kit (S0027, Beyotime Biotechnology, Shanghai, China) according to the manufacturer’s instructions. The luminescence signal was recorded using a Spark multimode microplate reader (Tecan, Grodig, Austria), and the values were normalized to the protein content.

### 2.14. ROS Quantification

The L4-stage nematodes were randomly transferred to plates containing 5 mg/mL CS and incubated for 5 d. Following that, nematodes were collected into centrifugal tubes with 50 μM 2′,7′-dichlorodihydrofluorescein diacetate (C3890-50, Apexbio, Houston, TX, USA) and incubated in the dark for 4 h. Nematodes were randomly placed on the slides for fluorescence imaging. ROS levels were quantified using ImageJ software to determine the fluorescence intensity.

### 2.15. Immunofluorescence Staining

Following treatment, nematodes were collected into centrifugal tubes containing 3 μM Rhodamine123 (C2008S, Beyotime Biotechnology, China), 5 μM MitoSOX Red (M36007, Thermo Fisher, Waltham, MA, USA), or 3 μM Rhod-2AM (R1244, Thermo Fisher, USA), and incubated for 2 h in the dark. Nematodes were randomly selected and mounted onto slides for fluorescence imaging. The levels of mitochondrion-related indicators were quantified using ImageJ software to determine the fluorescence intensity.

### 2.16. RT-Quantitative Polymerase Chain Reaction (qPCR) Assay

Nematodes at the L4 stage were treated with 2.5 or 5 mg/mL CS for 5 d. Total ribonucleic acid (RNA) was extracted using TRIzol reagent (410411, Ambion, Austin, TX, USA). Complementary deoxyribonucleic acid (cDNA) was synthesized using a Hifair^®^ III First-Strand cDNA Synthesis Kit (11139ES60, Yeasen Biotechnology, Shanghai, China). Real-time-qPCR was performed using SYBR green fluorescent dye in a CFX Connect Real-Time System (Bio-Rad Laboratories, Hercules, CA, USA). The relative gene expression was analyzed using the 2^−ΔΔCt^ method relative to *act-1*. The primer sequences (Sangon Biotech, Shanghai, China) are listed in Supplementary Table S1.

### 2.17. Western Blotting

The L4-stage nematodes were randomly transferred to plates containing 2.5, 5 mg/mL CS and incubated for 5 d. Total protein from at least 150 nematodes was extracted and quantified using bicinchoninic acid protein assay kits (BL521A, Biosharp, Beijing, China). Western blotting was performed as previously described [33]. The primary antibodies used for western blotting were as follows: phosphorylated AMP-activated protein kinase alpha subunit (p-AMPKα) (2535S, CST, Fayetteville, GA, USA) and SIRT1 (RM0498, Biodragon, Shanghai, China). Potein bands were visualized using an Amersham Imager 600 system (GE Healthcare Bio-Sciences AB, Tokyo, Japan). The levels of targeted protein were quantified relative to β-actin.

### 2.18. Cell Culture and Treatment

HEK293T cells were acquired from the American Type Culture Collection (ATCC, Manassas, VA, USA) and cultured in Dulbecco’s modified eagle medium (C3113-0500, Viva Cell, Shanghai, China) containing 10% fetal bovine serum (E600001, BBI, Shanghai, China), 100 U/mL penicillin-streptomycin (15140122, Gibco, Billings, MT, USA) in a humidified atmosphere containing 5% carbon dioxide at 37 °C.

### 2.19. Dual-Luciferase Reporter Gene Analyses of SIRT1 Transcriptional Activity

The SIRT1 promoter region was cloned upstream of the firefly luciferase gene in the reporter plasmid (GP-miRGLO; Supplementary Table S2). A separate plasmid expressing Renilla luciferase under a constitutive promoter (pGL4.75) served as an internal control to normalize the transfection efficiency and cellular variability. When HEK293T cells growth density reached 70–80%, cells were co-transfected with firefly luciferase reporter plasmid GP-miRGLO-SIRT1-promoter and *Renilla reniformis* luciferase control plasmid pGL4.75 at a 30:1 ratio using polyethyleneimine (PEI, 1 μg/μL, 23966-1, Polysciences, Warrington, PA, USA). After 16–24 h, the CS compounds were added, and the cells were cultured for 6 h. Luciferase activity was sequentially evaluated using a Dual-Luciferase Reporter Gene Assay System (Promega, E1960, Fitchburg, WI, USA) on a Spark multimode microplate reader (Tecan, Grodig, Austria).

### 2.20. Ultra-High Performance Liquid Chromatography-Q Exactive-Mass Spectrometry (UHPLC-QE-MS) for the Identification of CS

UHPLC-QE-MS analysis was performed using UHPLC-quadrupole/Orbitrap MS (UHPLC-Orbitrap Exploris 120 (Thermo Fisher, Waltham, MA, USA)) to identify the components of CS extract with an ACQUITY UHPLC BEH C_18_ (2.1 × 100 mm; 1.7 μm) column. The mobile phase involved a mixture of aqueous solution (eluent A) and acetonitrile (eluent B). The gradient elution procedure was as follows: 0–30 min, at 5% B-95% B. The flow rate was recorded as 0.3 mL/min, with the injection volume being 3 μL. The ionization parameters were set as follows: sheath gas flow rate of 35 arb; auxiliary gas volume flow 10 arb; ion transfer tube temperature 320 °C; spray voltage: 3.5 kV (positive ion), −3.0 kV (negative ion). The MS analysis was performed both in full-scan positive and negative ionization modes, covering a mass range of *m*/*z* 100–1500. The scanning mode: full MS/ddMS^2^, Orbitrap resolution was 120,000; meanwhile, the dd-MS^2^ resolution was 15,000. The chemical characterization data are provided in Supplementary Table S3.

### 2.21. Statistical Analysis

All data are presented as the mean ± standard error of mean (SEM) from at least three independent biological experiments. GraphPad Prism 8.0 and ImageJ were employed for all statistical analyses. Unpaired two-tailed Student’s *t*-tests were used to analyze the statistical significance between the two groups. The log-rank (Mantel–Cox test) test was utilized to analyze the statistical significance of the lifespan and stress resistance assays. A *p*-value < 0.05 was considered statistically significant.

## 3. Result

### 3.1. CS Extended the Lifespan and Improved the Health Status of C. elegans

**Figure 1:**
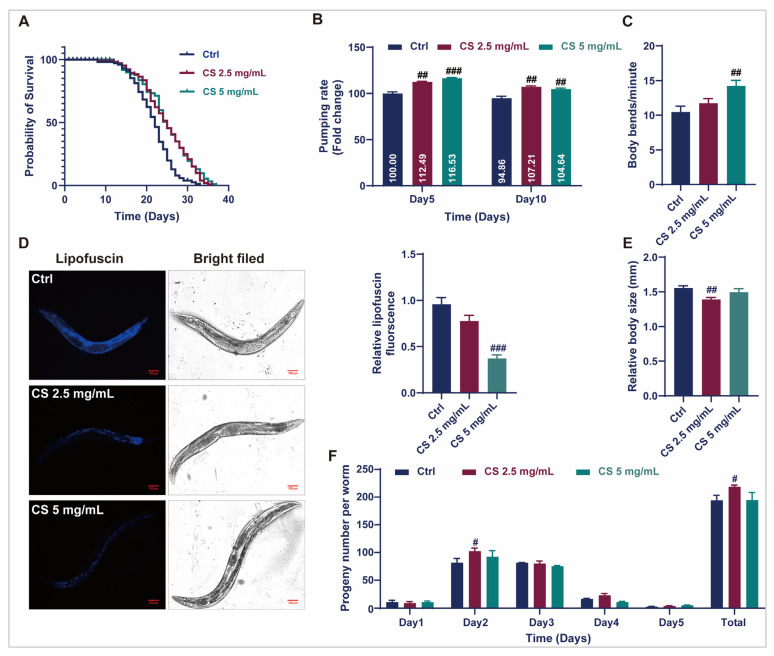
CS extended the lifespan and improved the physiological indices of *C. elegans*. (**A**) Effect of CS on the lifespan of *C. elegans*. (**B**) Effect of CS on the pharyngeal pumping frequency of *C. elegans* for 30 s. (**C**) Effect of CS on the locomotor ability of *C. elegans* for 60 s. (**D**) Effect of CS on the accumulation of lipofuscin in *C. elegans*. Scale bar = 100 μm. (**E**) Effect of CS on the body length of *C. elegans*. (**F**) Effect of CS on *C. elegans* reproduction. Data are shown as the mean ± SEM. # *p* ≤ 0.05, ## *p* ≤ 0.01, ### *p* ≤ 0.001.

To efficiently assess the anti-aging effects of CS, optimal concentrations for *C. elegans* were primarily established. As demonstrated in Supplementary Figure S1A, L4-stage *C. elegans* cultured with 0, 1.25, 2.5, and 5 mg/mL CS exhibited similar rates of food consumption, indicating that CS at concentrations between 1.25 and 5 mg/mL is not toxic to *C. elegans*. Therefore, CS concentrations of 2.5 and 5 mg/mL were selected for subsequent assays. As displayed in Figure 1A, CS concentrations of 2.5 and 5 mg/mL significantly prolonged the mean lifespan of N2 nematodes by 15.23% and 15.26%, respectively. Furthermore, compared to the control group, CS (5 mg/mL) markedly enhanced the frequency of pharyngeal pumps within 30 s in nematodes on the 5th and 10th d of adulthood by 16.53% and 4.64%, respectively (Figure 1B). CS treatment (5 mg/mL) markedly increased the number of body bends in N2 nematodes by 35.91% within 60 s on the 5th d of adulthood (Figure 1C). Additionally, the accumulation of lipofuscin, an oxidation by-product of lysosomal degradation, decreased to 38.54% when the nematodes were treated with 5 mg/mL CS (Figure 1D). The body length of wild-type *C. elegans* was also reduced following treatment with CS (2.5 mg/mL) (Figure 1E). Furthermore, the daily fecundity and total brood size rates were augmented by CS compared to those of untreated N2 nematodes (Figure 1F). Taken together, our data demonstrated that CS supplementation prolonged the lifespan of nematodes and improved their health status in N2 nematodes.

### 3.2. CS Protected Against Oxidative Stress in C. elegans

**Figure 2:**
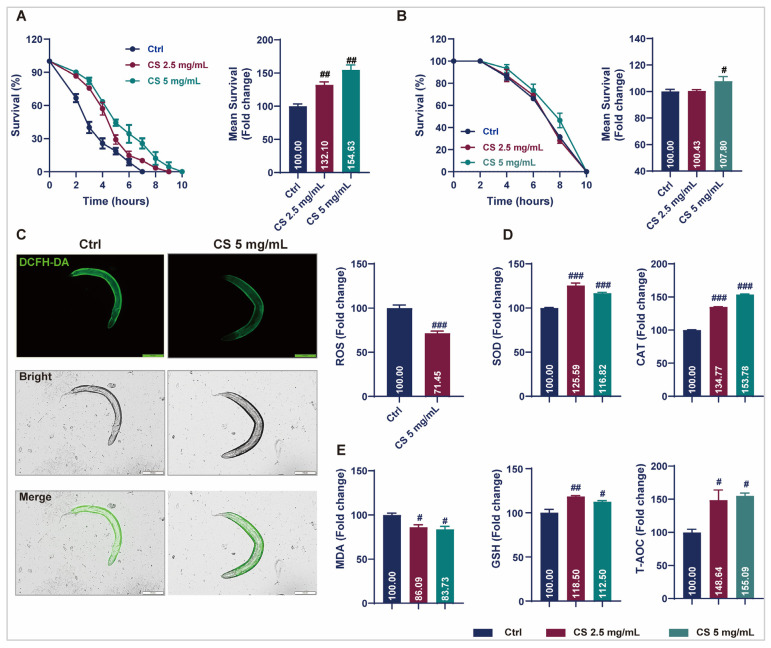
CS enhanced stress resistance and attenuated oxidant responses in *C. elegans*. (**A**) Effect of CS on the lifespan of *C. elegans* under H_2_O_2_ incubation. (**B**) Effect of CS on the lifespan of *C. elegans* under 37 °C heat stress. (**C**) Effect of CS on ROS accumulation in *C. elegans*. Scale bar = 200 μm. (**D**) Effect of CS on SOD, CAT, GSH and T-AOC enzyme activities in *C. elegans*. (**E**) Effect of CS on MDA production in *C. elegans*. Data are shown as the mean ± SEM. # *p* ≤ 0.05, ## *p* ≤ 0.01, ### *p* ≤ 0.001.

Prolonged longevity in nematodes is generally associated with enhanced stress resistance, as genes contributing to stress response signaling are often intertwined with longevity regulation [16]. Therefore, to investigate the potential of CS in modulating of stress endurance, the survival of nematodes exposed to oxidative stress or acute heat was assessed. Data revealed that CS increased the survival rate of nematodes under 2 mM H_2_O_2_-induced oxidative stress by 32.10% and 54.63% in responses to concentrations of 2.5 and 5 mg/mL, respectively (Figure 2A). After thermal stimulation at 37 °C, the average survival time of the nematodes increased by 7.80% when treated with 5 mg/mL CS (Figure 2B). ROS levels were determined, and CS treatment reduced intracellular ROS by 28.55% (Figure 2C). The activities of SOD, CAT, GSH, and T-AOC were determined. As anticipated, all the enzymatic activities were significantly increased after the addition of CS. Compared to the control group, 2.5 mg/mL and 5 mg/mL of CS increased SOD, CAT, GSH, and T-AOC activities to 125.59% and 116.82%, 134.77% and 153.78%, 118.50% and 112.50%, 148.64% and 155.09%, respectively (Figure 2D). Furthermore, the level of lipid peroxidation MDA was significantly reduced in nematodes following CS treatment (Figure 2E). These results suggested that the lifespan extension induced by CS may be associated with its antioxidative activity.

### 3.3. CS Maintained Mitochondrial Homeostasis and Activated Stress Response Pathways in C. elegans

**Figure 3:**
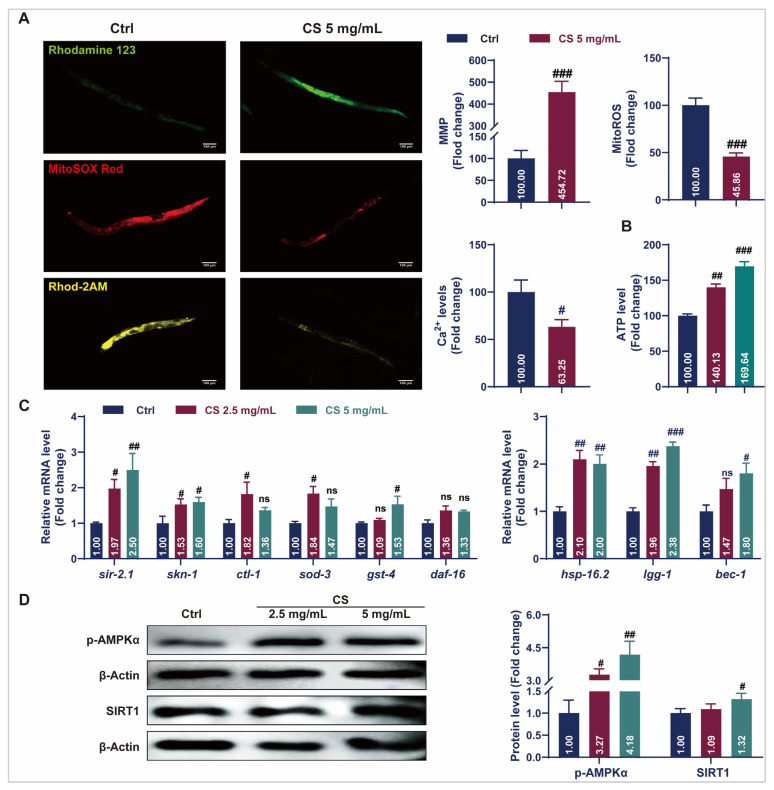
CS improved mitochondrial function and initiated protective mechanisms in *C. elegans*. (**A**) The representative fluorescent images and analyzed data of CS on the MMP, mitochondrial ROS, and mitochondrial Ca^2+^ levels in *C. elegans*. Scale bar = 100 μm. (**B**) Effect of CS on ATP content in *C. elegans*. (**C**) Effect of CS on anti-aging related gene expressions in *C. elegans*. (**D**) Effect of CS on the protein expressions of p-AMPKα and SIRT1 in *C. elegans*. Data are shown as the mean ± SEM. # *p* ≤ 0.05, ## *p* ≤ 0.01, ### *p* ≤ 0.001. ns (not statistically significant) indicates no significant difference between groups.

Aging causes mitochondrial dysfunction characterized by electron leakage from the respiratory chain and excess ROS production [34]. Fluorescent staining assays revealed that CS treatment increased the rhodamine123-labeled MMP, reduced MitoSOX red-labeled mitochondrial ROS levels, and decreased Rhod-2 AM-labeled mitochondrial calcium (Ca^2+^) levels (Figure 3A). Additionally, treatment with 2.5 or 5 mg/mL CS increased ATP content in *C. elegans* by 40.13% and 69.64%, respectively (Figure 3B). The expression of stress response genes in the nematodes was also investigated. As indicated in Figure 3C, compared to the control group, the messenger RNA (mRNA) levels of *sir-2.1*, *skn-1*, *clt-1*, *sod-3*, and *gst-4* were significantly up-regulated in the CS treatment groups. No significant differences were observed in the *daf-16* mRNA expression. Additionally, treatment with CS significantly up-regulated the expression of autophagy-related genes *lgg-1* and *bec-1* in *C. elegans*. Activation of the heat shock protein HSP-16.2 promoted the resistance of *C. elegans* to heat stress [19]. Consistently, the gene *hsp-16.2* was also up-regulated after CS treatment. AMP-activated protein kinase (AMPK)/SIRT1 activation plays a critical role in the regulation of aging and lifespan. In accordance, CS (5 mg/mL) significantly up-regulated the expression of p-AMPKα and SIRT1 by 4.18 and 1.53 times, respectively (Figure 3D). These findings indicated that CS prolonged the lifespan by improving mitochondrial function and activating stress response pathways in *C. elegans*.

### 3.4. Sir-2.1 Is Essential for the CS-Induced Extension of Lifespan and Enhancement of Oxidative Stress Resistance in C. elegans

**Figure 4:**
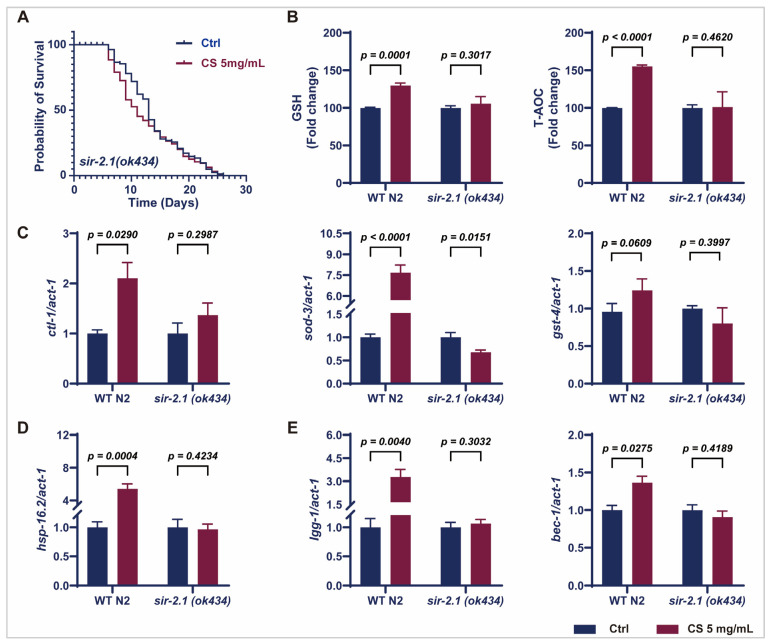
CS modulated SIR-2.1/SIRT1 to extend lifespan and resist oxidative stress in *C. elegans*. (**A**) Effect of CS on the lifespan of *sir-2.1(ok434)* mutant nematodes. (**B**) Effect of CS on GSH and T-AOC enzyme activities in WT N2 and *sir-2.1(ok434)* mutant nematodes. (**C**) Effect of CS on anti-oxidant related genes (*ctl-1*, *sod-3*, *gst-4*) expressions in WT N2 and *sir-2.1(ok434)* mutant nematodes. (**D**) Effect of CS on heat shock gene (*hsp-16.2*) in WT N2 and *sir-2.1(ok434)* mutant nematodes. (**E**) Effect of CS on autophagy-related genes (*lgg-1*, *bec-1*) expressions in WT N2 and *sir-2.1(ok434)* mutant nematodes. Data are shown as the mean ± SEM. WT: wild type.

Considering that CS increased sir-2.1/SIRT1 expression in N2 worms (Figure 3C,D), we further evaluated the effects of CS on knockout *sir-2.1 C. elegans*. No significant extension of the mean lifespan of *sir-2.1(ok434)* mutants was observed following CS treatment compared to that in the untreated control group (Figure 4A). Furthermore, CS increased the activity of GSH and T-AOC in wild-type N2 *C. elegans*; however, these effects were attenuated in the *sir-2.1(ok434)* mutants (Figure 4B). Moreover, no significant differences were noted in the mRNA expression of *ctl-1*, *sod-3*, and *gst-4* in *sir-2.1(ok434)* mutants between the control and CS administration groups (Figure 4C), whereas CS effectively up-regulated the expression of these genes in WT N2 nematodes. Similarly, CS treatment had little or no effect on the mRNA levels of *hsp-16.2*, *lgg-1*, and *bec-1* in *sir-2.1(ok434)* mutants (Figure 4D,E). These aforementioned data revealed that sir-2.1/SIRT1 was necessary for an extended lifespan and resistance to oxidative stress induced by CS.

### 3.5. Chemical Characterization of CS

**Figure 5:**
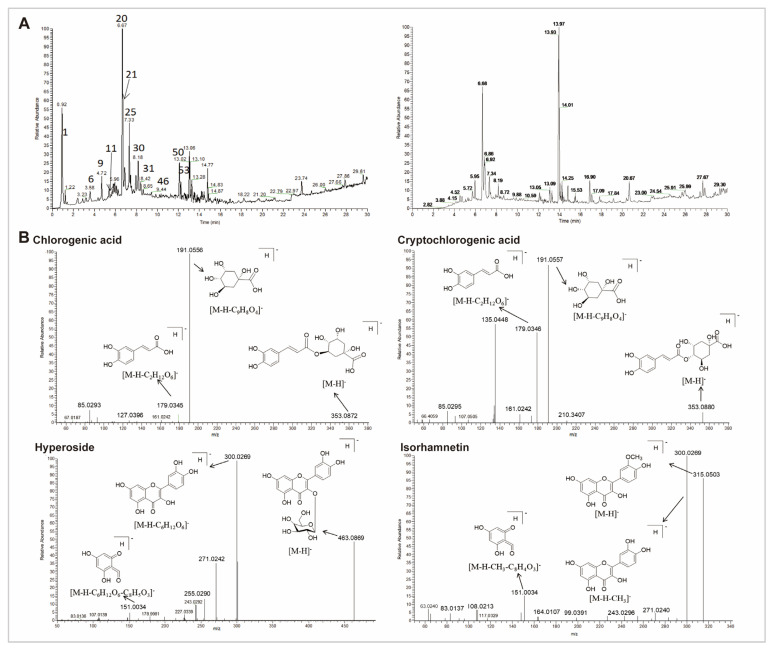
UHPLC-QE-MS of 70% ethanol extract from CS. (**A**) TIC in the negative (**left**) and positive (**right**) in electrospray ionization (ESI) mode. (**B**) The representative mass spectra and proposed cleavage pathway of chlorogenic acid, cryptochlorogenic acid, hyperoside, and isorhamnetin in CS.

The total ion chromatogram (TIC) of the CS extract in both positive and negative ionization modes is demonstrated in Figure 5A and Supplementary Table S3. Data revealed that 54 compounds were identified from CS extract, including 18 organic acids, 15 flavonoids, seven fatty acids, three alkaloids, seven glycoside acids, two lignans, and two other components. Pharmacological studies have demonstrated that the active components of CS primarily include phenolic acids and flavonoids [20,28]. Therefore, we particularly focused on organic acids and flavonoids. Based on the peak height, four organic acids (citric acid, caffeic acid, chlorogenic acid, and cryptochlorogenic acid), as well as three flavonoids (hypericin, isorhamnetin, and quercetin) were identified as the representative compounds for the cleavage pathway analysis (Figure 5B and Supplementary Figure S1B).

### 3.6. Active Compound of CS Enhanced the Transcriptional Activity of SIRT1 in HEK293T Cells

**Figure 6:**
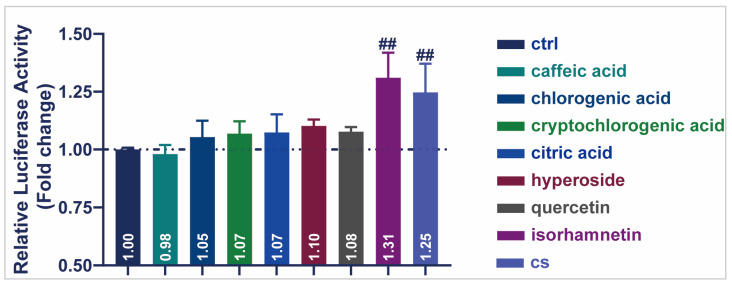
Effects of CS and seven compounds from CS on SIRT1 transcriptional activity. Effect of representative compounds of CS on SIRT1 transcriptional activity. Data are shown as the mean ± SEM. ## *p* ≤ 0.01.

A dual-luciferase activity assay was performed to detect the transcriptional activity of seven representative compounds of CS (Figure 6). All other compounds enhanced SIRT1 transcriptional activity in HEK293T cells, except for caffeic acid (0.98). Notably, isorhamnetin produced the highest luciferase activity (1.31), indicating the up-regulation of SIRT1 transcription. These results demonstrated that the monomeric components of CS, especially isorhamnetin, increased the transcriptional activity of SIRT1 and played a role in delaying aging.

## 4. Discussion

Although aging is irreversible, identifying potential candidates to delay its progression and extend healthy lifespan remains a critical goal. In traditional Chinese medicine, CS has been recognized as a key ingredient capable of slowing the natural aging process in mice [25,27]. In the present study, we discovered that CS extended the lifespan of *C. elegans* by suppressing SIRT1-mediated oxidative stress and mitochondrial dysfunction.

*C. elegans* serves as an ideal model for exploring the process of aging [6,7,18]. Despite the lack of key physiological systems and the simplicity of transcriptional regulation in nematodes, physiological dysregulation resulting from the molecular events of aging has been extensively studied [16,32]. More than 500 compounds with anti-aging properties have been identified in the DrugAge database, most of which have been tested using nematodes [35]. Based on our data, CS prolonged the lifespan (Figure 1A) and increased the bending and pumping rates in *C. elegans* (Figure 1B,C), demonstrating the anti-aging activity of CS in nematodes. Physical integrity and fertility are two essential indicators of longevity in *C. elegans* [36,37]. In the present study, CS significantly reduced nematode body length (Figure 1E) and enhanced offspring production, as evidenced by a significant increase in the total brood size (Figure 1F).

Aging is closely related to redox balance in the body [4]. As aging progresses, the antioxidant defense system deteriorates, resulting in the accumulation of excessive ROS and increased oxidative stress. Lipofuscin is an oxidative byproduct of lysosomal degradation, and its accumulation is regarded as a marker of aging [38]. Consistently, we identified that CS prolonged the survival of nematodes in response to H_2_O_2_ (Figure 2A) and heat stress (Figure 2B), reduced ROS content (Figure 2C), and decreased lipofuscin accumulation (Figure 1D), indicating that the antioxidative properties of CS contribute to its anti-aging effects of CS in *C. elegans*. Furthermore, a series of antioxidant enzymes, including SOD, CAT, GSH, and T-AOC, were detected, and as expected, CS increased antioxidant enzymatic activities (Figure 2D). Moreover, MDA, is a common marker of lipid peroxidation [39], was significantly reduced by CS treatment in nematodes (Figure 2E), consistent with the previous finding in D-galactose-induced aging mice administered with CS [40].

Mitochondria are the primary organelles involved in oxidative stress and energy metabolism [41]. With nematode aging, the structure and function of mitochondria gradually degenerate, resulting in MMP reduction, ROS accumulation, and Ca^2+^ overload [16,26,42]. Conversely, mitochondrial homeostasis is recognized as a marker of longevity, as evidenced in centenarians and long-lived species [43,44]. This is accompanied by a reduction in ATP synthesis and an insufficient energy supply for cells, which affects various physiological behaviors, including motility and reproduction. These are typical manifestations of nematode aging [45,46]. Consistent with previous reports, our results demonstrated that treatment with CS resulted in increased levels of MMP and ATP, while decreasing mitochondrial ROS and Ca^2+^ levels (Figure 3A,B), providing evidence that mitochondria were targeted by CS to prevent of oxidative stress during the anti-aging process in *C. elegans*.

Longevity depends on the synergistic action of numerous signaling pathways to maintain cellular homeostasis [16]. Notably, SIR-2.1/SIRT1 is an important metabolic sensor closely related to mitochondrial biogenesis [41], which synergizes with AAK-2/AMPK to ameliorate mitochondrial dysfunction via the suppression of oxidative stress [41]. Recently, Todorova et al. reported that AAK-2/AMPK and SIR-2.1/SIRT1 were the primary targets of the natural medicine *Punica granatum* L. leaf extract for enhancing stress resistance during nematode aging, with these effects significantly diminished in *aak-2* and *sir-2.1* deficient *C. elegans* [16]. Consistent with this finding, we noted that supplementation with CS extract activated the phosphorylation of AAK-2/AMPK and increased the expression of SIR-2.1/SIRT1 (Figure 3D). Additionally, *clt-1*, *sod-3*, and *gst-4* are known to be positively regulated by SIR-2.1/SIRT1, while the interplay modulates ROS production and oxidative stress injury through the regulation of the transcriptional activity of SKN-1/NRF2 [8,47]. Our results align with these findings, as treatment with CS resulted in increased expression of *clt-1*, *sod-3*, and *gst-4*, suggesting the involvement of the SIRT1-NRF2 axis in conferring CS-mediated resistance to oxidative stress. This is further supported by the up-regulation of the canonical regulator of the oxidative stress response, *skn-1*, of which the overexpression is associated with oxidative stress inhibition [17]. In addition, the longevity of *C. elegans* is promoted by extra copies of the *sir-2.1* gene in a manner dependent on the forkhead transcription factor *daf-16* [48]. However, no obvious response was observed for *sir-2.1*-dependent *daf-16* expression following CS treatment in *C. elegans* (Figure 3C), indicating a selective mechanism for CS-mediated SIR-2.1/SIRT1 regulation. One possible interpretation is that *sir-2.1* influences lifespan in a *daf-16*-independent manner as part of the caloric restriction pathway [14,49]. Moreover, autophagy is up-regulated in nematode longevity mutations (*daf-2*, *eat-2*, *glp-1*, *rsks-1*, and *clk-1*) [50]. Upstream regulators of autophagy include AMPK, SIRT1, and NRF2, all key determinants of longevity [51,52]. Indeed, the PCR data of N2 nematodes suggested that CS significantly induced the expression of the autophagy genes *lgg-1*, and *bec-1*, illustrating that the CS-induced improvements in lifespan and mitochondrial function may require autophagy. Additionally, HSP-16.2, the representatives of the HSP protein family, was used as a predictor of longevity in *C. elegans*, as it is closely related to heat tolerance and longevity [18]. Evidence suggests that heat shock proteins promote increased survivability and fitness in a *sir-2.1*-dependent manner [53]. We discovered that CS improved the resistance of *C. elegans* to heat stress (Figure 2B), and further, PCR revealed that CS played a role by up-regulating the expression of the heat stress-related gene *hsp-16.2* (Figure 3C). These observations emphasized the close relationship between SIRT1 and the mechanism by which CS delayed aging in nematodes, further supported by related experiments on CS in *sir-2.1(ok434)* mutant nematodes (Figure 4A–E).

Studies have demonstrated that the potential anti-aging benefits of polyphenols and flavonoids are garnering scientific interest owing to their ability to modulate oxidative damage, inflammation [54,55,56]. The chemical characterization of CS extract demonstrated that it was mainly composed of phenolic acids and flavonoids (Figure 5A and Supplementary Table S3), consistent with the findings of previous studies [20,28]. According to the method proposed by Baker et al., a dual-luciferase reporter gene system was established in HEK293T cells (a human renal epithelial cell line) to preliminarily and rapidly screen for effective components of CS that regulated SIRT1 [57]. Consequently, isorhamnetin in CS extract significantly enhanced the transcriptional activity of SIRT1 (Figure 6), which is consistent with the outcomes of Wu’s study [58]. Therefore, isorhamnetin may be an effective compound in CS extract for delaying aging in *C. elegans*.

Regretfully, although this study confirmed that the sir-2.1 pathway is necessary for the action of CS, the possibility that CS synergistically affects the phenotype by regulating other genes cannot be ruled out. Specific targets need to be fully analyzed and validated by transcriptome sequencing and nematode mutants. This study only evaluated the effect of monomeric compounds on the transcriptional activity of SIRT1 in vitro; however, the protein stability of SIRT1 may affect its final function. Further comprehensive assessments are required to combine protein and enzyme activity tests in *C. elegans*.

## 5. Conclusions

**Figure 7:**
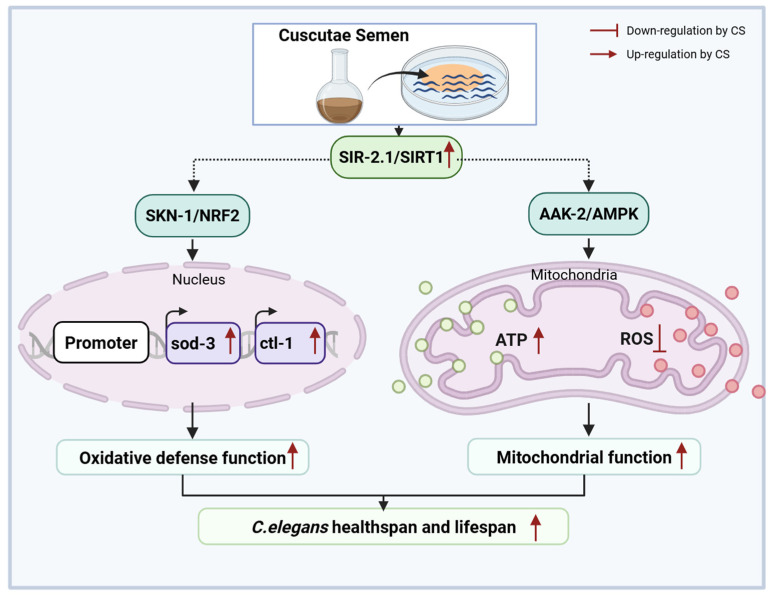
Molecular mechanisms of CS in delaying aging in *Caenorhabditis elegans*.

In conclusion, CS extended *C. elegans* lifespan and ameliorated mitochondrial oxidative stress via SIRT1, indicating that CS is a promising candidate for improving longevity (Figure 7).

## Data Availability

The data analyzed during the study are freely available upon request from the corresponding authors.

## Supplementary Materials

The following supporting information can be downloaded at: https://www.mdpi.com/article/10.3390/antiox14070786/s1. References [59,60,61,62,63,64,65,66,67,68,69,70,71] are cited in the supplementary materials.

## Abbreviations

CS	Cuscutae Semen
*C. elegans*	*Caenorhabditis elegans*
MMP	Mitochondrial membrane potential
ATP	Adenosine triphosphate
NGM	Nematode growth medium
SOD	Superoxide dismutase
CAT	Catalase
GSH	Glutathione
T-AOC	Total antioxidant capacity
SIRT1	Silent information regulator sirtuin 1
p-AMPKα	Phosphorylated AMP-activated protein kinase α subunit
NRF2	Nuclear factor erythroid 2-related factor 2
FOXO	Fork-head box
ESI	Electrospray ionization
UPLC	Ultra-performance liquid chromatography
MS	Mass spectrometry
TIC	Total ion chromatogram
