# Supplementary material for: Herbal Cuscutae Semen Contributes to Oxidative Stress Tolerance and Extends Lifespan via Sirtuin1 in Caenorhabditis elegans
Source: Antioxidants (Basel). 2025 Jun 26;14(7):786. doi: 10.3390/antiox14070786 (PMC12291695; doi:10.3390/antiox14070786)
Supplement: Supplementary file 1 [file antioxidants-14-00786-s001.zip › antioxidants-3649378-supplementary.pdf]

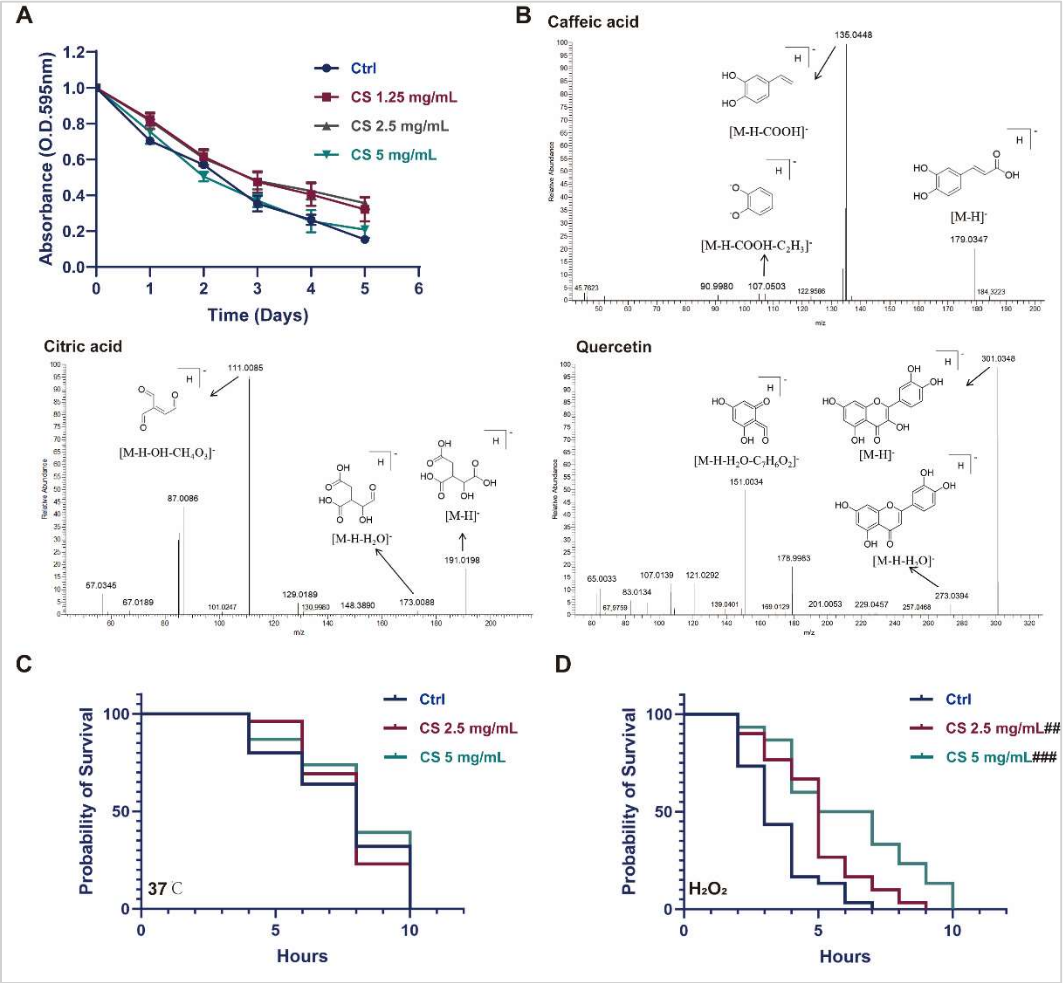

Supplementary Figure S1. (**A)** Effect of CUs on food clearance in *C. elegans.* Data are shown as mean ± SEM. **(B)** The representative mass spectra and proposed cleavage pathway of caffeic acid, citric acid, and quercetin in CS. (**C)** The change in survival rate of *C. elegans* exposed to heat shock at 37°C. **(D)** The change in survival rate of C. elegans exposed to H_2O_2-induced oxidation.

Supplementary Table S1. Primer used for RT-qPCR.

Target gene	gene accession number	Forword primer sequence	Reserve primer sequence
sir-2.1	NM_001268555	TTCGTGTGGTTCGTCTTGCTCATC	TCCATCACATGAATCGGCTCGTTG
skn-1	NM_171347	TAGCCGACGACGAAGAAGAGACG	ATTGGATTGAGGTGTTGGACGATGG
ctl-1	NM_064578	CCGTACTCGTGATGATGTGAAGGAG	GCTGCTCGTAGTTGTGATCGTCTC
sod-3	NM_078363	ATCACTATTGCGGTTCAAGGCTCTG	TTGCACAGGTGGCGATCTTCAAG
gst-4	NM_069447	AGTTGTTGAACCAGCCCGTGATG	CAGCCCAAGTCAATGAGTCTCCAAC
daf-16	NM_001377628	CCACCACCATCATACCACGAGTTG	CATTGGCTTGAAGTTAGTGCTTGGC
hsp-16.2	NM_001392482	TGTAGATGTTGGTGCAGTTGCTTCG	CTTCGACGATTGCCTGTTGAATTGG
lgg-1	NM_062876	GTGGGCTTACAAGGAGGAGAACAAC	GGAATACGGTCTGGGTACTTTCTGC
bec-1	NM_068443	CTGTCAGCATCCGTTGAGGTTGG	GGTTTCGAGAATGTCCAGTCAGAGC
act-1	NM_073418	CTGTCCTCTCCCTCTACGCTTCC	CAGTAAGATCACGTCCAGCCAAGTC

Supplementary Table S2. Sequence of the human SIRT1 promoter (5'-3', 2101bp)

TGTCAACCACTAGGAGTGTGGTGCCTAGTCAGGAATTGGGAGGAGTGTAGCAAGAAAGGAAGGACAACAGGATT
TGGTCATTGATTGGTCAGATGGATTTCAGAGGGATTGGTATGAAGGAACGCTTCAAAGATTTTTTTTTAATTTAAG
TTCCAGGATACAGGTGCAGAATGTGTAGGTTTGTTACATACTTATAGGTGAGCCATGGTGGTTTGCTGCACCAATCA
ACCCCTCATCTAGGTTTTATTTATATTTTTTTTTGAGACGGAGTTTCGCTCTTGCTGCCCAGGCTGGTGTGCAATGGT
GCGATCTCGGCTCACCGCAACCTCTGCCTGCTGGGTTCAAGCGATTCTCCTGCCTCAGCCTTCTGAGTAGCTGGGA
TTACAGGCATGTGCCACCACGCCCGGCTAATTTTGTATTTTTAGTAGAGACGGGGTTTCTCCATGTTGGTCAGGCTG
GTCTCGAACTTCCGACTTCAGGTGATCTGTCCGCCTCGGCCTCCCAAAGTGCTGGGATTACAGGCAGCAGCCACC
GCGCCCAGCCAGGTTTTAAGTCCCGCATGCATTAGCTATTTGTCCTAATGCTCTCCCTCCCCTTGACCCCCACGCAA
CCAAAGATGGTTTTAAGGCTTCTAGGACTGGAGATGATTACTTTCAGAAATGAATAGTGGTGACCAGATGAAATAA
GACTTTTGGATGTAGATAGGACCTTAGCGATATATTTCCAGCTTTTTATTTTTTATTTTAAGGATGAAGAAACAGCTT
TAAGTTGGTTGCCTAAAGTCACGCAGGTAATTGATGCAGTATGGCCAGAACCCATACTAGGCTTAAGGGACAGTGT
ACCATAAACTCGTAGAGGTCCTTTAAACAGATAGAAACGCTGTGCTCCAGGCAGATGCCATAACAAACACTGGCT
CTAGATCTACCATGGGTTATATGGGTCCTTAGTTAAGATTAGATATGGAGTCACAGTGTGCCAGAATTTCAGGGAGA
GAGGAAAGTGGAAGGGCTTTCCACTAAGCCTTTTGAACTACTAGGTACCCCTCGTTTTACATCTGGTTATCTCATTT
AAATCTATGACGTTTTAAAATACTTATTACCATTTAAGACATGAGAAAAATTAAGTTTAGAAACGGCTAGATAGCTC
ACGCTAGAAAGGAAGGACTCCAAATTTTAACCAAGGGCAGATGTGCATGGAGGCCAAGTCATTTCCTTCCCATGC
TCTCATACTGACCCAACAAACCCATTCTGCACGTGAGAAAACTGAGGCCCGGAGGAGGGAATTCACACACGTTTG
AAGCCAAGCTGGGGCCAGAAAGTAGATCGGCTGATCTCCAAACCTCCACGTCAAAGGTCTTCCCAGGAGGACATA
TGCCTTCAAGGATTTTACAATGTATACCACCCTACAAGTGATGGGAGAGAGGGGAAAAAAGCAACCGACTAAGGA
GAAAAGCAAGGAGCAGAAAAAGGAGCAAAAGAGGAGCTGTCAGAACGGTGTGAGGAGAGTGGGAAAGGAGCC
GCCTCCTTTTGCCTCTCTTCCTACTTATTAACAAAACAGAACGACTATCCAACGTATTTCAGGGAGCTAAGTCTTAG
CCAGCTTCAGCTGTGTTTTAACCCTTAGCTAAATATAGACAAGGCTAAGGCAGGCCAGGTGTACACTTCAGGAAGA
CGTGGAAATTCCCAGGGCGGACCAAAACTTGAGCTGTTCCGGCGGTAGTGATTTGAGGTCAGTTTGAAAGAGAA
GTTGAGAAAGCGGCCGAGGGGCGAATTTGGCTGCACTACACGCTCGCCACAAAGAGGAAGGGCCGCCGGCCGCC
GGGGCCGAGTGCGCTTCCAGCCCAGGCGGAGCGGTAGACGCAACAGCCTCCGCCCGCCACGTGACCCGTAGTGT
TGTGGTCTGGCCCGCGTGGGTGGCGGGAGCGCCGAGAGGGCGGGGGCGGCGATGGGGCGGGTCACGTGATGGG
GTTTAAATCTCCCGCAGCCGGAGCCGCGGGGGCGCCAGTGCCGCGCGTCGAGCGGGAGCAGAGGAGGCGAGGG
AGGAGGGCCAGAGAGGCAGTTGGAAGATGGCGGACGAGGCGGCCCTCGCCCTTCAGCCCGG

Supplementary Table S3. Monomeric compounds in CS extracts.

ID	Compound name	Rest Time (min)	Theoretical value	Measure value	Ion mode	ESI-MS^2	M.W.	Type	ref
1	citric acid*	1.00	191.0192	191.0198	[M-H]-	173.0088、129.0189、111.0085	192.027	Organic acid	/
2	isocitric acid	1.20	191.0192	191.0194	[M-H]-	173.0085、129.0188、111.0085	192.027	Organic acid	[1]
3	3-*O*-caffeoylquinic acid-glucoside or isomer	3.21	515.1401	515.1398	[M-H]-	353.0868、191.0567、179.0345	516.1479	Organic acid	[2]
4	3-*O*-caffeoylquinic acid-rhamnoside or isomer	3.28	515.1401	515.14	[M-H]-、[M+H]+	191.0560、173.0455、163.0392	516.1479	Organic acid	[2]
5	maltol*	3.49	127.0395	127.0385	[M+H]+	115.9065、109.0280、81.0333	126.0317	Other	/
6	cryptochlorogenic acid*	3.63	353.0867	353.0880	[M-H]-	191.0557、179.0346、135.0448	354.0951	Organic acid	/
7	quercetin-3-*O*-galactosyl-7-*O*-glucoside	4.58	625.1399	625.1396	[M-H]-、[M+H]+	463.0873、301.0349、151.0032	626.1483	Flavonoid	[3]
8	chlorogenic acid*	4.72	353.0867	353.0878	[M-H]-、[M+H]+	191.0556、161.0242、85.0293	354.0951	Organic acid	/
9	quinic acid	4.74	191.0556	191.0556	[M-H]-	173.0448、127.0399、85.0294	192.0634	Organic acid	/
10	caffeic acid*	5.37	179.0338	179.0351	[M-H]-	179.0347、135.0448	180.0423	Organic acid	/
11	6-*O*-(trans)-*p*-Coumaroyl-*β*-D-fructofuranosyl-(2→1)-*α*-D-glucopyranoside	5.37	487.1446	487.1449	[M-H]-	341.1101、325.0930、163.0396	488.1530	Other	[4]
12	kaempferol-3,7-di-*O*-*β*-D-glucopyranoside	5.38	609.1456	609.1445	[M-H]-	447.0922、285.0398、151.0036	610.1534	Flavonoid	[3]
13	cis-4-*O*-coumaroylquinic acid	5.74	337.0923	337.0936	[M-H]-	293.0008、191.0556、173.0450	338.1002	Organic acid	[5]
14	trans-4-O-coumaroylquinic acid	5.90	337.0923	337.0934	[M-H]-	292.9249、191.0562、173.0451	338.1002	Organic acid	[5]
15	4-caffeoyl-5-coumaroylquinic acid-diglucoside	6.10	823.2297	823.2286	[M-H]-	661.1765、499.1284、191.0556	824.2375	Organic acid	[3]
16	5-*O*-Feruloylquinic acid	6.27	367.1024	367.1016	[M-H]-	282.3237、191.0556、173.0452	368.1107	Organic acid	[5]
17	cis-4-coumaric acid*	6.69	163.0395	163.0396	[M-H]-	119.0500、93.0345	164.0473	Organic acid	/
18	quercetin-3-*O*-apiosyl-(1→2）-galactoside	6.87	595.1293	595.1293	[M-H]-、[M+H]+	301.0344、271.0241、151.0034	596.1377	Flavonoid	[6]
19	cuscutalin A	6.95	239.0815	239.0820	[M-H]-、[M+H]+	221.0715、195.0923、180.0689	240.0899	Alkaloid	[7]

20	hyperoside*	7.33	463.0871	463.0869	[M-H]-、[M+H]+	301.0346、271.0242、151.0034	464.0955	Flavonoid	[8]
21	kaempferol-coumaroyl-glucoside or isomer	7.82	755.1823	755.1818	[M-H]-	593.1290、447.0934、285.0399	756.1902	Flavonoid	[6]
22	kaempferol-3-*O*-galactoside	7.90	447.0922	447.0927	[M-H]-、[M+H]+	327.0510、284.0321、151.0036	448.1006	Flavonoid	[5]
23	dihydrophaseic acid or isomer	8.06	281.1389	281.1389	[M-H]-	239.1489、219.1382、171.1174	282.1467	Organic acid	[9]
24	pinoresinol 4-glucopyranoside	8.13	519.1866	519.1865	[M-H]-	357.1337、151.0398、136.0163	520.1945	Lignan	[6]
25	astragalin*	8.18	447.0921	447.0937	[M-H]-、[M+H]+	284.0321、227.0346、151.0036	448.1006	Organic acid	/
26	isorhamnetin-3-*O*-*β*-D-glucoside(iso）*	8.22	477.1028	477.1031	[M-H]-、[M+H]+	314.0426、271.0243、151.0036	478.1111	Flavonoid	/
27	isorhamnetin-3-*O*-*β*-D-glucoside*	8.35	477.1028	477.1032	[M-H]-、[M+H]+	314.0426、271.0243、151.0034	478.1111	Flavonoid	/
28	isochlorogenic acid C*	8.67	515.1184	515.1194	[M-H]-	353.0869、191.0554、179.0345	516.1268	Organic acid	/
29	azelaic acid	8.79	187.0973	187.0965	[M-H]-	141.8678、125.0969、97.0657	188.1043	Fatty acid	[1]
30	hydroxybenzoate-quercetin-3-*O*-galactoside	8.94	583.1088	583.1081	[M-H]-、[M+H]+	463.0863、301.0343、151.0031	584.1166	Flavonoid	[6]
31	kaempferol-3-*O*-*p*-hydroxybenzoylglucoside	9.40	567.1133	567.1134	[M-H]-、[M+H]+	447.0926、284.0320、227.0345	568.1217	Flavonoid	[6]
32	4-caffeoyl-5-coumaroylquinic acid	9.69	499.1240	499.1241	[M-H]-	353.0841、191.0555、179.0342	500.1319	Organic acid	[5]
33	cuscutamine	9.70	269.0920	269.0929	[M-H]-	253.4878、225.1029、156.0815	270.1004	Alkaloid	[10]
34	4-feruloyl-5-caffeoylquinic acid	9.75	529.1346	529.1345	[M-H]-	367.1032、191.0565、173.0452	530.1424	Organic acid	[3]
35	querccetin-3-*O*-coumaroylgalactoside	9.89	609.1239	609.1239	[M-H]-	463.0872、301.0346、151.0036	610.1323	Flavonoid	[6]
36	7'-(3',4'-hydroxyphenyl)-[4-(methoxyphenyl)ethyl]acrylamide	9.92	312.1236	312.1233	[M-H]-、[M+H]+	297.0988、190.0500、178.0505	313.1314	Alkaloid	[7]
37	quercetin*	10.55	301.0342	301.0348	[M-H]-	229.0497、301.0352、178.9984	302.0427	Flavonoid	/
38	cuscutoside A	11.28	663.1919	663.1924	[M-H]-	369.0970、340.0955、219.0660	664.2003	Lignan	[11]
39	cuscutic acid A3	11.69	713.3601	713.3587	[M-H]-	567.3019、405.2488、243.1962	714.3674	Glycoside acid	[11]
40	apigenin*	11.83	269.045	269.0457	[M-H]-、[M+H]+	225.0561、151.0035、107.0344	270.0528	Flavonoid	/

41	kaempferol*	12.13	285.0399	285.0399	[M-H]-	257.0347、239.0347、151.0034	286.0477	Flavonoid	/
42	12,13,16-trihydroxyoctadeca-9,14-dienoic acid or isomer	12.27	327.2169	327.2171	[M-H]-	291.1961、229.1439、211.1335	328.2250	Fatty acid	[1]
43	isorhamnetin*	12.35	315.0499	315.0505	[M-H]-、[M+H]+	300.0269、271.0240、151.00334	316.0583	Flavonoid	/
44	12,13,17-trihydroxyoctadeca-9-enoic acid or isomer	13.01	329.2322	329.2325	[M-H]-	293.2123、211.1335、171.1229	330.2406	Fatty acid	[1]
45	cuscutic acid A2	13.01	697.3655	697.3638	[M-H]-	679.3503、551.065、243.1962	698.3725	Glycoside acid	[11]
46	cuscutic acid B	13.03	829.4069	829.4056	[M-H]-	577.3268、51.1306、243.1961	830.4137	Glycoside acid	[11]
47	cuscutic acid C	13.09	843.4226	843.4267	[M-H]-	577.3133、351.1280、243.1959	844.4304	Glycoside acid	[11]
48	cuse3	14.61	797.4171	797.4173	[M-H]-	753.3887、679.3541	798.4249	Glycoside acid	[12]
49	cuse1	16.98	839.4277	839.4261	[M-H]-	621.2755、276.5316、243.1960	840.4355	Glycoside acid	[13]
50	cuse2	17.14	869.4746	869.4714	[M+H]+	563.9897、483.3325	868.4668	Glycoside acid	[13]
51	(11S)-jalapinolic acid or isomer	20.50	271.2273	271.2272	[M-H]-	253.2161、225.2230、109.7598	272.2351	Fatty acid	[1]
52	hydroxyoctadece dienoic acid	21.22	295.2273	295.2283	[M-H]-	277.2167、171.1021、155.1073	296.2351	Fatty acid	[1]
53	(11S)-jalapinolic acid or isomer	26.29	271.2273	271.2271	[M-H]-	253.2167、255.2218、197.1905	272.2351	Fatty acid	[1]
54	linoleic acid	27.85	279.2322	279.2325	[M-H]-	243.7905、206.8245、134.8943	280.2402	Fatty acid	[1]

"*" Indicates identification by comparison of the reference material.

[1] Pubchem. (2025). Compound database [Dataset]. Retrieved from https://pubchemncbinlmnihgov.

[2] Zong Z, Hu Y, Xu L.-L, et al. Based on UPLC-Q-TOF-MS and network pharmacology, the components and mechanisms of Cuscuta chinensis-Lycium barbarum in the treatment of early-onset ovarian insufficiency were explored[J]. Chinese Traditional Patent Medicine, 2024, 47(02): 649-658.

[3] Li W, Zhang Y, Bai J, et al. Identification of chemical constituents in Cuscuta chinensis using HPLC-ESI/Q-TOF MS/MS[J]. Bio Technol Indian J, 2013, 8(4): 563-567.

[4] Gao T.-H. Studies on chemical constituents of Cuscuta chinensis Lam[D], Jilin: Jilin University, 2009.

[5] Zhang Y. A Comparative Study on the Chemical Components and Tonifying Kidney Yang Effects of Different Processed Products of Cuscuta chinensis[D]. Beijing: Beijing University of Chinese Medicine, 2018.

[6] Ye M, Yan Y N, Guo D A. Characterization of phenolic compounds in the Chinese herbal drug Tu-Si-Zi by liquid chromatography coupled to electrospray ionization mass spectrometry[J]. Rapid Commun Mass Spectrom, 2005, 19(11): 1469-1484.

[7] Anis E, Anis I, Ahmed S, et al. Choudhary M I. α-glucosidase inhibitory constituents from Cuscuta reflexa[J]. Chem Pharm Bull, 2002, 50(1): 112-114.

[8] Li G.-W, Sun D.-M, Hu Q.-P, et al. Fingerprints and quantitative analysis of Cuscuta australis R. Br. processed products[J]. Central South Pharmacy, 2022, 20(05): 992-998.

[9] Wang J, Tan D, Wei G, et al. Studies on the Chemical Constituents of Cuscuta chinensis. Chem Nat Compd, 2016, 52(6): 1133-1136.

[10] Rho T, Yoon K D. Application of off-line two-dimensional high-performance countercurrent chromatography on the chloroform-soluble extract of Cuscuta auralis seeds. J Sep Sci, 2018, 41(10): 2169-2177.

[11] Du X M, Kohinata K, Kawasaki T, et al. Components of the ether-insoluble resin glycoside-like fraction from Cuscuta chinensis. Phytochemistry, 1998, 48(5): 843-850.

[12] Fan B.-Y, Luo J.-G, Gu Y.-C, et al. Unusual ether-type resin glycoside dimers from the seeds of Cuscuta chinensis. Tetrahedron, 2014, 70(11): 2003-2014.

[13] Vijikumar S, Ramanathan K, Devi B. Cuscuta reflexa Roxb-A wonderful miracle plant in ethnomedicine. Indian J Nat Sci, 2011, 976: 997.
